# Before the diagnosis: quality of life in patients presenting with chest pain undergoing Bruce protocol stress testing

**DOI:** 10.1186/s12872-025-05281-8

**Published:** 2025-10-29

**Authors:** Ghassan Mourad, Pallav Deka, Dola Pathak, Raquel Lopez-Vilella, Leonie Klompstra, Elena Marques-Sule

**Affiliations:** 1 Department of Health, Medicine and Caring Sciences, Linköping University, Kungsgatan 40, Norrköping, SE-601 74 Sweden; 2 College of Nursing, Wayne State University, Detroit, MI USA; 3 Department of Statistics and Probability, Michigan State University, East Lansing, MI USA; 4 Servicio de Cardiologia, Unidad de Insuficiencia Cardíaca y Trasplante, Hospital Universitari i Politècnic La Fe, Valencia, Spain; 5 Physiotherapy in Motion. Multispeciality Research Group (PTinMOTION), Department of Physiotherapy, University of Valencia, Valencia, Spain

**Keywords:** Chest pain, Non-cardiac chest pain, Stress test, Quality of life

## Abstract

**Background:**

A stress test is commonly used as an initial evaluation tool for determining a possible cardiac origin for chest pain. This study was performed to test the hypothesis that patients with a positive stress test will have a significantly lower health-related quality of life (HRQoL) than patients with a negative stress test.

**Methods:**

This study was a two-group non-experimental design where group assignment was based on the result of the stress test. Participants completed an HRQoL questionnaire (EuroQol-5D) prior to performing the Bruce treadmill stress test. The stress test was considered positive if participants reported chest pain or developed electrocardiogram ST-T changes or arrhythmia during the test.

**Results:**

A total of 123 participants completed the study with 33 participants with a positive stress test and 90 participants with a negative stress test. Participants with a negative stress test reported a significantly lower (*p* = 0.014) HRQoL as compared to participants with a positive stress test. In the overall sample, current smokers (*p* < 0.001) and women (*p* = 0.02) were found to have a lower HRQoL.

**Conclusions:**

Patients with non-cardiac chest pain can experience lower HRQoL. The HRQoL was not related to results of the diagnostic testing. This study underscores that patients with non-cardiac chest pain could benefit from additional psychological assessment and treatment.

## Background

Stable angina, also referred to as exertional or exercise-induced angina, is indicative of coronary ischemia when perfusion demands of the myocardium during physical exertion or stress are not met due to atherosclerotic stenosis [1]. Along with chest pain with or without radiation, patients with coronary artery disease (CAD) also commonly report tachycardia, palpitation, shortness of breath, nausea, diaphoresis, and fatigue [2]. However, chest pain is not always cardiac related. In fact, in many instances, patients with musculoskeletal, gastrointestinal, pulmonary, and psychological problems may also report chest pain with associated symptoms that are indistinguishable from ischemic cardiac pain [3, 4]. In the clinical setting, a treadmill exercise stress test, using the Bruce protocol, is a commonly used non-chemical tool for the initial evaluation of patient-reported chest pain and for ruling out a non-cardiac from a cardiac etiology [5]. A positive Bruce stress test is indicated by the patient reporting chest pain, and/or exhibiting electrocardiogram (EKG) changes or underlying arrhythmias during the test [6–8]. Future medical management is based on the outcome of the test being positive or negative.

Studies have indicated that patients diagnosed with CAD experience a lower health- related quality of life (HRQoL) [9, 10]. The prevalence of chest pain from non-cardiac reasons [11] and HRQoL post-completion of a diagnostic test for CAD has been documented [12]. The study reported that patients with non-cardiac chest pain with a history of cardiac disease experienced a significantly lower QoL as compared to patients with no cardiac disease [12]. Evaluation of Qol in that study was done after patients had knowledge of their diagnosis, although both patients with previous CAD and those without had presented with chest pain of non-cardiac origin at the time for the evaluation. There is reasons to believe that the knowledge of a disease diagnosis can alter perceptions of health and QoL. For example, there is evidence in the cancer population that knowledge of cancer diagnosis can impact perceived sense of control [13]. Also, health perception and lifestyle alterations have been seen post diagnosis in patients with hypertension [14]. These examples are supported by the Theory of Unpleasent Symptoms that indicates that symptom experience is multifactorial and is the result of a complex interaction between physiological, psychological and situational factors [15]. Furthermore, previous research has demonstrated a positive association between HRQoL and several cardiovascular risk factors. Specifically, HRQoL was positively correlated with male sex, active aging, lower body mass index (BMI), and non-smoking status [16–21]. However, not much has been reported about QoL in patients experiencing chest pain prior to performing a diagnostic test. Hence, the purpose of this study was to investigate if patients with a positive stress test (indicative of potential CAD) experience lower HRQoL than patients with a negative stress test. Additionally, we also aimed to investigate the effect of cardiovascular risk factors of sex, age, BMI, current smoking status on HRQoL.

## Methods

### Design

This study was a two-group non-experimental cross-section design where group assignment was based on the result of the stress test. The study was approved by the Human Ethics Committee at the University of Valencia. Informed consent was obtained prior to participation and data were collected between January 2013 and December 2015. All procedures were carried out in compliance with the Declaration of Helsinki.

### Sample and setting

All participants enrolled in the study had complaints of chest pain and were recommended the treadmill stress test by their cardiologist during regular visit to the cardiology clinic at the University of Valencia. Participants were excluded if they had unstable angina, severe aortic stenosis, uncontrolled hypertension, and were unable to walk on a treadmill.

### Outcome variables and measures

(i) Quality of Life: The Spanish validated version of the EuroQol Visual Analogue Scale (EQ-VAS) was used to measure HRQoL [22, 23]. The EQ-VAS provides a single quantitative measure of general self-rated health on a vertical visual analogue scale ranging from 0 (worst imaginable health state) to 1 (best imaginable health state). An overall Cronbach’s alpha of 0.74 has been found for the tool in patients with CAD [24].
(ii) Positive/negative stress test: The Bruce protocol [25] was used for the stress test and recommendations for exercise testing were followed [26]. Participants were asked to walk on a treadmill and the workload was increased every 3 min by increasing the pace and grade. Theoretically, the test can last a maximum of 21 min with a maximum treadmill speed of 5.5 mph and 22% grade. Participants were asked to take their beta-blocker as usual but were asked not to eat, drink or smoke for 3 h prior to the test to enable the patient to achieve a higher workload. A resting EKG and blood pressure were monitored at the end of each stage of the test. The test was stopped if any of the following outcomes were noticed: The outcome responses for symptoms and signs we specifically monitored included: (i) patient-reported chest pain; (ii) patient-reported breathlessness/exhaustion and not being able to keep up with test protocol; (iii) EKG changes specifically for ST changes or arrhythmias; and (iv) patients heart rate (HR) reaching their target HR_max_ defined as reaching 85% of age-predicted HR_max_ (220 - age). Additionally, we recorded any other reasons why patients may not have completed the tests.

### Procedure

Patients were recruited during their scheduled visit to the clinic for their scheduled stress test. They were screened for inclusion and exclusion criteria and research personnel obtained informed consent and provided details of the procedure and the QoL questionnaire. Participants were asked to take their medications as directed by the cardiologist, be adequately hydrated and not perform any exercise on the day of the test. Participants completed the QoL questionnaire before the stress test. Help was provided to complete the questionnaire if needed. Data were collected by the same staff on all participants. The stress test was stopped if any of the above-mentioned outcomes of the test were achieved or for any other reason (for example, participant not being able to walk on a treadmill). Demographic and clinical characteristics (Table 1) of the consented participants were obtained from participants’ medical records.

**Table 1:** Sociodemographic and clinical characteristics

	Positive stress test (*n* = 33)	Negative stress test (*n* = 90)
Age years, mean ± SD	60.5 ± 9.3	59.1 ± 9.9
Sex, n (%)		
Male	28 (84.85%)	71 (78.89%)
Female	5 (15.15%)	19 (12.22%)
BMI (kg/m^2^), mean ± SD	29.0 ± 3.97	29.1 ± 3.62
Previous coronary revascularization, n (%)		
PCI	17 (51.52%)	37 (41.11%)
CABG	1 (3.03%)	11 (12.22%)
Drugs, n (%)		
Antiplatelets	32 (96.97%)	88 (97.78%)
Beta-blockers	30 (90.91%)	76 (84.44%)
ACE Inhibitors	16 (48.47%)	42 (46.67%)
ARBs	6 (18.18%)	11 (12.22%)
CCB	3 (9.09%)	8 (8.89%)
Diuretics	2 (6.06%)	3 (3.33%)
Statins	29 (87.88%)	82 (91.11%)
CVRF, n (%)		
Hypertension	21 (63.64%)	67 (74.44%)
Dyslipidemia	29 (87.88%)	82 (91.11%)
Diabetes	8 (24.24%)	26 (28.89%)
Current smoker	2 (6.06%)	10 (11.11%)
Previous Smoker	24 (72.73%)	63 (70.00%)

*Abbreviations*: *ACE *Angiotensin-converting enzyme, *ARBs *Angiotensin II receptor blockers, *BMI *Body mass index, *CABG *Coronary artery bypass grafting, *CCB *Calcium channel blockers, *CVRF *Cardiovascular risk factors, *PCI *Percutaneous coronary intervention

### Analysis

Descriptive statistics including mean, standard deviation, and percentage were used for describing demographic characteristics, clinical characteristics, and outcome measures. We created two groups of participants with a positive or negative stress test. An independent t-test was done to determine any significant differences in QoL outcomes between the two groups’ risk factors of CAD (i.e., sex, age, BMI, current smoking status). If equality of variance assumption was not met for the data, a Welsch t-test was used for the analysis [27]. Statistical significance was set at alpha of 0.05. Statistical analysis was performed with R software 4.1.0.

## Results

**Fig. 1:**
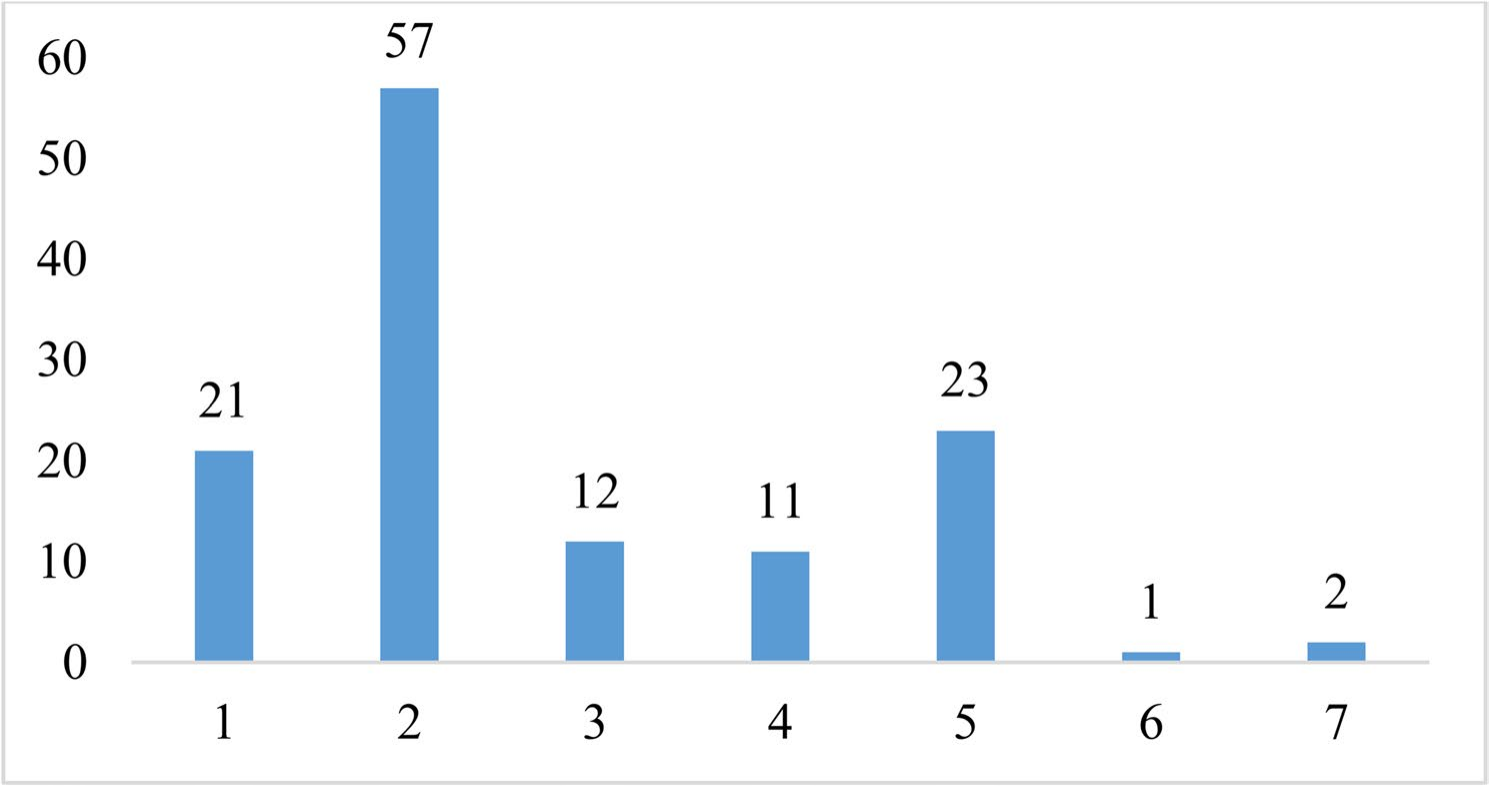
Bruce treadmill stress test outcomes. Labels: 1 = EKG changes; 2 = Breathlessness/Shortness of breath; 3 = Chest pain; 4 = Leg pain/claudication; 5 = Reach HR_max_; 6 = Dizziness; 7 = Did not adapt to treadmill

A total of 127 patients participated in the study. One participant reported feeling dizzy during the test and 3 participants were not able to adjust to walking on a treadmill. Excluding these 4 participants, the final analysis included 123 participants who completed the both stress test and the EQ-5D questionnaire. During the stress test, 33 participants (27%) had a positive stress test by either demonstrating EKG changes (*n* = 21) during the test or reporting chest pain (*n* = 12) (Fig. 1). EKG abnormalities mostly included ST changes (*n* = 20) with one patient having arrhythmias. Some patients (*n* = 11) reported leg pain (knee or ankle pain) or claudication-type pain during the stress test but were able to complete the test. The remaining 90 (73%) had a negative stress test.

The demographic and clinical characteristics of the number of participants with a positive or negative stress test are presented in Table 1.

Table 2 shows the results of an independent t-test in QoL between patients who tested positive and negative in the stress test. As the equality of variance assumption was not met for the data, a Welsch t-test was used for the analysis.

**Table 2:** HRQoL compared with the stress test result

Stress test outcome	Positive test (*n* = 33)	Negative test (*n* = 90)	t score	*p*-value
HRQoL (mean ± SD)	0.86 ± 0.13	0.78 ± 0.22	−2.48	0.014

*Abbreviations*: *HRQoL *Health-related quality of life

Effect of the interaction between the groups and sex/age/BMI/current smoking status on QoL was found to be non-significant. As such, the overall effect of BMI, sex and smoking status on QoL was assessed. In the overall sample, a significant difference in HRQoL was seen between current smokers and non-smokers (*p* < 0.001) and between men and women (*p* = 0.02). Current smokers and women were found to have a lower HRQoL. Functional capacity as determined by Metabolic Equivalents (METS) was not significantly different between the positive stress test group (mean METS 7.88 ± 2.62) and the negative stress test group (mean METS 7.15 ± 2.15).

## Discussion

Our study findings were contrary to our expectations. Although HRQoL was reasonably high in both groups, patients with a negative stress test who were likely experiencing chest pain of non-cardiac origin reported significantly lower HRQoL compared to those with a positive stress test, even before the test was performed. This may be explained by the fact that participants completed the HRQoL questionnaire prior to knowing their test results. Additionally, patients with non-cardiac chest pain often lack a clear diagnosis or explanation for their symptoms despite multiple healthcare encounters, which can lead to increased anxiety, uncertainty, and psychological distress—factors known to negatively affect perceived quality of life. However, it is important to acknowledge the large difference in sample size between the positive stress test group (*n* = 33) and the negative stress test group (*n* = 90), and its impact on power and generalizability of the results of this study. Future research should be done in this area with larger and similar sample sizes to account for difference in variances between the two groups.

Patients with a positive stress test will likely receive further cardiac intervention in the form of percutaneous coronary intervention needing stents or coronary artey bypass grafting depending on the extent of the disease. These patients are also likely to receive a subsequent referral to cardiac rehabilitation post-intervention which has been shown to significantly improve quality of life, functional status, anxiety, and depression in patients with CAD [28, 29]. Likewise, a negative stress test with non-cardiac origin (psychological, musculoskeletal or gastrointestinal related) also warrants further evaluation. It is documented that patients with non-cardiac chest pain may be discharged without a clear explanation of the underlying cause of their chest pain [30–33] and without any planned follow-up for psychological, musculoskeletal, pulmonary, or gastrointestinal issues [34, 35]. Previous research has also found that depending on cardiologist evaluation, patients with a negative stress test experiencing chest pain may or may not receive medication management (with antianginals) or an invasive diagnostic test [36]. This is an area where close collaboration between the cardiologist office and primary care provider becomes important.

Previously, it has been reported that there is no difference in anxiety and depression among patients who report anginal pain either for a cardiac or non-cardiac etiology [37]. In our study, participants completed the HRQoL questionnaire prior to performing the stress test and the mental distress may be for two reasons. Firstly, for patients with history of cardiac diseases, their anxiety and distress may be in anticipation of another diagnosis of CAD possibly needing hospitalization and intervention. Secondly, for patients with no cardiac history, anxiety may be related to not yet having a diagnosis for the recurrent chest pain that they are experiencing. Also, while patients with a positive stress test from underlying coronary atherosclerosis and stenosis are likely to experience chest pain with exertion, patients experiencing chest pain from a non-cardiac etiology, most notably from either pshycological, musculoskeletal or gastrointestinal conditions, can experience chest pain even without exertion contributing to the experience of a lower HRQoL. These patients often report dissatisfaction with not having a good explanation for their condition and simply having a “ruled out” diagnosis [32, 33]. As supported by the Theory of Unpleasent Symptoms such physiological and psychological states along with being in a situation of not having a diagnosis can impact overall health [15]. Nurse practiotioners, especially in primary care, have a special role to play in management of care for these patients especially in promoting self-care behaviors including physical activity, diet and sleep habits.

Patients experiencing chest pain often show reduced engagement in physical activity and lower QOL which can be attributed to kinesiophobia or underlying psychiatric disorders [38–41]. The fear may be a protective mechanism of preventing self-harm or injury but can cause long-term avoidance of physical activity [42]. Avoidance behaviour can lead to maintenance and exacerbation of fear and pain [43, 44]. This can in turn lead to disability, impaired quality of life, cardiac-related anxiety, increased chest pain, and high and inappropriate use of medical care [41, 45–47]. The societal cost associated with the treatment of non-cardiac chest pain can be significantly higher than the cost of treating cardiac chest pain [48]. These patients may also frequently seek care for psychological reasons [49]. As such, beyond traditional practices of reassurance, [50] and to reduce healthcare utilization, primary care providers and cardiologists should be mindful of the decline in QoL in patients with non-cardiac chest pain and provide appropritate referral for assessment and treatment of the underlying reason for pain. Once chest pain has been adequately managed, engagement in physical activity and exercise should be encouraged to improve functional status, anxiety, depression, and overall HRQoL. Adequate social support from healthcare providers and family will also be essential for these patients for improving self-efficacy and improving HRQoL [51]. 

In our study, we did not find any significant differences in the cardiovascular risk factors (sex, age, BMI, current smoking status, METS) between the positive and negative stress test groups. Consistent with the literature, in the overall sample, our study did find smoking status to be significantly associated with HRQoL with current smokers reporting a lower HRQoL [17]. Healthcare providers should continue to stress the importance of smoking cessation in patients with both cardiac and non-cardiac chest pain. Additionally, we did not find any sex-related HRQoL differences based on the result of the stress test. We had a larger percentage of men than women in our population which may have impacted our results and studies on patients with established CAD have shown lower HRQol among women than men [18] – [19] Future intervention studies should design strategies to address this concern.

Our study was cross-sectional and included a sample recruited in Spain with a larger percentage of men than women. Pain can be multifactorial and can be influenced by not only physiological processes but also psychological attributes such as anxiety, depression, expectation, attention, [52] culture and race [53]. As such, any generalization of the results should be made cautiously. We only used one QoL measure and a similar study using other QoL measures with a larger sample should be done to validate our findings.

## Conclusions

HRQoL in patients with non-cardiac chest pain may be lower than in patients with cardiac chest pain. The HRQoL was not related to results of the diagnostic stress testing. This study underscores that patients with non-cardiac chest pain could benefit from additional psychological assessment and treatment. Primary care providers should make appropriate specialist referral for patients with non-cardiac chest pain and, after adequately controlling pain, provide encougarement for participation in physical activity and exercise to improve overall QoL. As women and active smokers tend to experience a lower QoL, special consideration should be given to these subgroups of patients who report chest pain that may be of cardiac or non-cardiac origin.

## Data Availability

The datasets used and/or analysed during the current study are available from the corresponding author on reasonable request.

## Abbreviations

CAD: Coronary artery disease
EKG: Electrocardiogram
EQ-5D: EuroQol-5D
HR: Heart rate
HRQoL: Health-related quality of life
METS: Metabolic Equivalents
